# Supplementary material for: Identification of photoperiod-induced specific miRNAs in the adrenal glands of Sunite sheep (Ovis aries)
Source: Front Vet Sci. 2022 Jul 22;9:888207. doi: 10.3389/fvets.2022.888207 (PMC9354845; doi:10.3389/fvets.2022.888207)
Supplement: Supplementary file 1 [file Data_Sheet_1.ZIP › Supplementary file/miRDeep2 file.zip!/miRDeep2 file/Novel_Structure/LP42A3/Novel_93.pdf]

Provisional ID	:	NC_019464.2_10007
Score total	:	2.4
Score for star read(s)	:	-1.3
Score for read counts	:	0
Score for mfe	:	2.1
Score for randfold	:	1.6
Score for cons. seed	:	
Total read count	:	19
Mature read count	:	16
Loop read count	:	0
Star read count	:	3

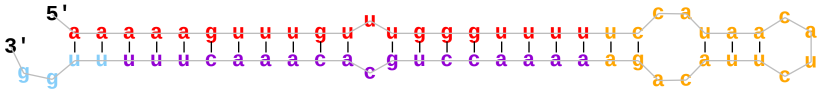

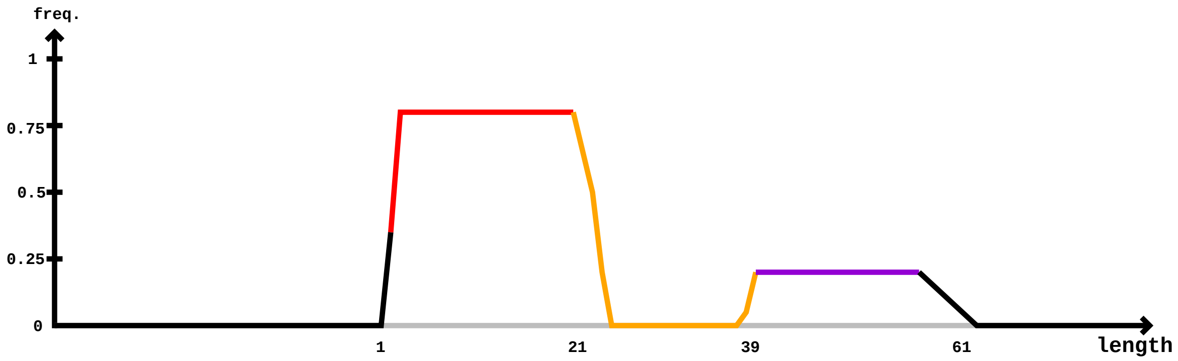


```
                                       Mature                                       Star
5'- ggcucauuaaaaugcuggagcauugaguuggccaaaaaguuuguuuggguuuuuccauaacaucuuacagaaaaaccugcacaaacuuuuuggccagcccaacauguagccc   -3'    obs
    ggcucauuaaaaugcuggagcauugaguuggccaaaaaguuuguuuggguuuuuccauaacaucuuacagaaaaaccugcacaaacuuuuuggccagcccaacauguagccc          exp
    (((((((...(((((....)))))(.((((((((((((((((((.((((((((((..(((....)))..)))))))))).)))))))))))))))))).)...))).)))).   reads   mm   sample
    .................................aaaaaguuuguuCggguuuu...........................................................   4       1    seq
    .................................aaaaGguuuguuuggguuuuuc.........................................................   1       1    seq
    .................................aaaaaguuuguuuggguuuuucc........................................................   2       0    seq
    ..................................Gaaaguuuguuuggguuuuu..........................................................   1       1    seq
    ..................................aaaGguuuguuuggguuuuu..........................................................   1       1    seq
    ..................................Gaaaguuuguuuggguuuuuc.........................................................   2       1    seq
    ..................................aCaaguuuguuuggguuuuuc.........................................................   1       1    seq
    ..................................aaaGguuuguuuggguuuuuc.........................................................   1       1    seq
    ..................................aaaaguuuguuuggguuuuuU.........................................................   1       1    seq
    ..................................Gaaaguuuguuuggguuuuucc........................................................   2       1    seq
    ......................................................................aaaaaccugAacaaacuuuu......................   1       1    seq
    .......................................................................aaaaccugAacaaacuuu.......................   2       1    seq
    .......................................................................aaaaccugAacaaacuuuu......................   1       1    seq
```
